# Factors associated with research participation in a large primary care practice-based pediatric cohort: Results from the TARGet Kids! longitudinal cohort study

**DOI:** 10.1371/journal.pone.0284192

**Published:** 2023-04-11

**Authors:** Xuedi Li, Charles D. G. Keown-Stoneman, Cornelia M. Borkhoff, Peter D. Wong, Dana Arafeh, Erika Tavares, Sharon Thadani, Jonathon L. Maguire, Catherine S. Birken, on behalf of the TARGet Kids! Collaboration

**Affiliations:** 1 Child Health Evaluative Sciences, The Hospital for Sick Children, Toronto, Ontario, Canada; 2 Li Ka Shing Knowledge Institute, St. Michael’s Hospital, Unity Health Toronto, Toronto, Ontario, Canada; 3 Dalla Lana School of Public Health, University of Toronto, Toronto, Ontario, Canada; 4 Division of Paediatric Medicine, The Hospital for Sick Children, Toronto, Ontario, Canada; 5 Institute of Health Policy, Management and Evaluation, University of Toronto, Toronto, Ontario, Canada; 6 Department of Pediatrics, Temerty Faculty of Medicine, University of Toronto, Toronto, Ontario, Canada; 7 Department of Family and Community Medicine, Temerty Faculty of Medicine, University of Toronto, Toronto, Ontario, Canada; 8 Patient Partner, Toronto, Ontario, Canada; 9 Department of Pediatrics, St. Michael’s Hospital, Unity Health Toronto, Toronto, Ontario, Canada; University of Adelaide School of Medical Sciences: The University of Adelaide Adelaide Medical School, AUSTRALIA

## Abstract

**Background:**

All longitudinal cohort studies strive for high participant retention, although attrition is common. Understanding determinants of attrition is important to inform and develop targeted strategies to improve study participation. We aimed to identify factors associated with research participation in a large children’s primary care cohort study.

**Methods:**

In this longitudinal cohort study between 2008 and 2020, all children who participated in the Applied Research Group for Kids (TARGet Kids!) were included. TARGet Kids! is a large primary care practice-based pediatric research network in Canada with ongoing data collection at well-child visits. Several sociodemographic, health, and study design factors were examined for their associations with research participation. The primary outcome was attendance of eligible research follow-up visits. The secondary outcome was time to withdrawal from the TARGet Kids! study. Generalized linear mixed effects models and Cox proportional hazard models were fitted. We have engaged parent partners in all stages of this study.

**Results:**

A total 10,412 children with 62,655 total eligible research follow-up visits were included. Mean age at enrolment was 22 months, 52% were male, and 52% had mothers of European ethnicity. 68.4% of the participants attended at least 1 research follow-up visit. Since 2008, 6.4% of the participants have submitted a withdrawal request. Key factors associated with research participation included child age, ethnicity, maternal age, maternal education level, family income, parental employment, child diagnosis of chronic health conditions, certain study sites, and missingness in questionnaire data.

**Conclusions:**

Socioeconomic status, demographic factors, chronic conditions, and missingness in questionnaire data were associated with research participation in this large primary care practice-based cohort study of children. Results from this analysis and input from our parent partners suggested that retention strategies could include continued parent engagement, creating brand identity and communication tools, using multiple languages and avoiding redundancy in the questionnaires.

## Introduction

In epidemiological research, the longitudinal cohort study is a powerful research design and offers numerous advantages by following a group of individuals over a period of time [1]. However, attrition, or loss of participants over the course of a study, is an inevitable occurrence in all longitudinal cohort studies and may compromise the power and validity of study findings and introduce bias due to differential loss to follow-up [2]. In order to develop strategies to reduce loss to follow-up and improve participant retention, it is crucial to identify factors associated with research participation.

Numerous pediatric longitudinal cohorts have studied research participation using a descriptive approach by comparing the characteristics of participants who continued in the study and those not followed up [3–9]. Compared to children who remained involved in the study, those lost to follow-up were more likely to be older children, male, and ethnic minority groups, had higher birthweights, had younger and foreign-born mothers, were less likely to live together with both parents, and had lower socioeconomic status [3–9]. Several longitudinal cohort studies in children examined the determinants of attrition by using a more rigorous analytical method and found that sociodemographic factors including older children, younger mothers, low parental educational level, single parenthood, and parents born in a foreign country were associated with higher attrition [10–13]. In addition, children with overweight or obesity, children with low well-being, and smoking and drinking in adolescents were associated with poor study participation [10, 11, 14]. Missingness (non-response items) at baseline has also shown to be positively associated with loss to follow-up, suggesting that individuals who did not participate fully at baseline were less likely to participate in future follow-ups [10]. Among these cohorts examining attrition determinants, two were school-based cohorts [10, 11, 14], one was a birth cohort, [15] and two cohorts focused on children at increased risk of medical conditions [12, 13]. No prior studies have examined factors associated with participation in healthy children recruited from a primary care setting.

Launched in 2008, the Applied Research Group for Kids (TARGet Kids!) [16] is a large primary care practice-based research network for children in Canada which recruits families with children under 5 years from primary care practices and invites participants to complete questionnaires and collect physical measures at each participant’s annual well-child visit. Identifying factors associated with research participation in the TARGet Kids! cohort study would help to inform and develop targeted strategies to improve participant retention in TARGet Kids!, but also other healthcare based cohort studies for children. Moreover, results from this study and our experience with participant retention during the past 12 years may provide valuable information to other researchers when designing and implementing future cohort studies. The objective of this paper was to determine sociodemographic, health, and study design factors associated with attendance of research follow-up visits and withdrawal from the TARGet Kids! longitudinal cohort study. We also aimed to summarize our current engagement strategies and outline opportunities of improvement. We hypothesized that various factors, including but not limited to, low socioeconomic status, parents not born in Canada, and missing questionnaire data would be associated with lower level of research participation.

## Materials and methods

### Study design and population

In this longitudinal cohort study, all children and their parents/caregivers participating in TARGet Kids! [16] enrolled between June 2008 and March 2020 (prior to the start of the COVID-19 pandemic) were included. TARGet Kids! (www.targetkids.ca) is a primary care practice-based research network for children in Canada, with over 100 primary healthcare providers from 15 large primary care practices participating across the Greater Toronto Area, Kingston, and Montreal [16]. The overall goal of TARGet Kids! is to improve the health of Canadians by optimizing growth and development through preventive interventions in early childhood. Since 2008, TARGet Kids! has been enrolling healthy children from primary healthcare practices and inviting them to participate at follow up well-child visits through adolescence [16]. At each regularly scheduled well-child visit, parents/caregivers of participating children were invited by a mailed letter to participate in a research follow up visit and to complete an age-specific standardized questionnaire adapted from the Canadian Community Health Survey [17] with questions on socio-demographic information, physical and mental health, health behaviours (e.g., nutrition, screen time, physical activity, and sleep), school and childcare arrangement, health services use, and developmental screening [16]. In addition to parent-reported questionnaire data, anthropometric data (child and parent height/length, weight, and waist circumference) were measured by a trained research assistant and families were invited to participate in non-fasting blood sample collection by a trained phlebotomist at each practice site [16]. Children were recruited at their well-child visit anytime between 0 to 5 years old [16]. All parents were invited by mail to participate with data collection twice a year before age 2 years, and then every year until age 18 years [16]. Research assistants would then approach participants who attended their well-child visit on site and invite them to participate. Children with health conditions affecting growth, children with chronic health conditions at enrolment except for asthma, children with severe developmental delay, and families who were not able to communicate in English or French were excluded from the TARGet Kids! cohort study [16]. Written consent was obtained from parents/caregivers of all participating children. This study was approved by the Research Ethics Boards at The Hospital for Sick Children (#10000–12436) and St. Michael’s Hospital (#17–335). The design of this study has been informed by input from our parent partners [18].

### Sociodemographic, health, and study design factors

Sociodemographic, health, and study design factors were identified from the published literature and classified as non-time-varying factors or time-varying factors. Data on none-time-varying factors were collected through parent-report questionnaires at enrolment and included child sex, maternal and paternal ethnicity, maternal education, maternal age at enrolment, biological mother and father place of birth, child immigration status, language spoken most often at home, maternal and paternal employment status, child birthweight, and TARGet Kids! practice site at enrolment. Employment status was only collected at enrolment and thus it was treated as a non-time-varying factor. Certain TARGet Kids! sites had few participants or shorter involvement with TARGet Kids! and therefore were grouped with larger sites.

Data on time-varying factors were repeatedly collected at each visit and data at the closest time point prior to each research follow-up visit were used, which represents the most recent information available to the research team. Time-varying factors included child age at each visit, self-reported annual family income, number of siblings, child living arrangement, dwelling type, food insecurity within the past year, child weight status, parent weight status, parent history of chronic health conditions, child history of chronic health conditions, and months of the well-child visit. Child age and month of the well-child visit were estimated based on the expected visit date in the case of non-visits. Each child’s height and weight were measured at each attended visit by trained research assistants at each site using standardized protocols from National Health and Nutrition Examination Survey (NHANES) [19]. Child weight was measured using an infant scale for children <2 years of age, and a calibrated precision digital scale for older children (Seca, Hamburg, Germany, www.seca.com/en_us/products/all-products.html). Child length was measured using a length board for children <2 years of age and standing height was measured using a stadiometer (Seca, Hamburg, Germany, www.seca.com/en_us/products/all-products.html). Height and weight of the parent who accompanied their child at the visit were also measured by trained research assistants. Body mass index (BMI) was calculated by dividing weight in kilograms by height (or length) in meters squared. Child BMI was age- and sex-standardized into zBMI scores using the World Health Organization Child Growth Standards, [20] as recommended in Canada [21].

All other time-varying factors were collected through repeated parent-reported questionnaires at each well-child visit. Food insecurity (Yes/No) was categorized as “No” if they answered “Never True” for the following two questions, otherwise food insecurity was categorized as “Yes”: Within the past 12 months we worried whether our food would run out before we got money to buy more (Never true/ Sometimes true/ Often true); Within the past 12 months the food we bought just didn’t last and we didn’t have money to get more (Never true/ Sometimes true/ Often true). Parent history of chronic health conditions (Any/None) was categorized as “Any” if the mother or the father of the child has been diagnosed with at least one of the following conditions: multiple sclerosis, diabetes, osteoporosis, heart disease, hypertension, high cholesterol, cancer, asthma, depression or anxiety, stroke, alcohol or drug problems, attention deficit hyperactivity disorder (ADHD), autism spectrum disorder (ASD), learning disability, overweight or obesity. Child history of chronic health conditions (Any/None) was categorized as “Any” if the child has been diagnosed with at least one of the following conditions: ADHD, allergies, asthma, ASD, developmental delay, diabetes, eczema, learning problem, overweight, cancer, or inflammatory bowel disease.

For time-varying factors, we used data at the closest time point before each research follow-up visit when examining their associations with study participation. When these factors were missing for the closest time point, data was used from the last visit of the same subject (forward filling). When no data was available to fill in, these factors were coded as a separate category “Missing” for non-response. Some of the factors we examined were not included in the early versions of the TARGet Kids! questionnaires. For example, annual family income was not asked in 2008; food insecurity was not asked before 2013; dwelling type was not asked between 2011 and 2013. For these questions, a separate category “Question not asked” was assigned, to differentiate from missingness. These approaches were chosen over other methods, such as imputation, as the variables in the model represent the information that would be available to a research team at that point in time, and not necessarily the true values of those variables if they had been successfully measured.

### Measures of research participation

The primary outcome of this study was attendance of eligible research follow-up visits, measured dichotomously (attended/missed). Attendance of a research follow-up visit was defined as the return of the parent-reported questionnaire for that visit. The number of eligible research follow-up visits per child was determined based on the date of enrolment, child age at enrolment, site stop date (some sites stopped working with TARGet Kids! during the study period), and withdrawal status. The secondary outcome of this study was time to withdrawal. Withdrawal from the study occurred when a parent submitted a request to withdraw their child from TARGet Kids!. Data collected on the child up to the point of withdrawal remained in the study database. When feasible, reasons for withdrawal were collected by the research assistants at each site.

### Statistical analysis

Participant characteristics were generated using descriptive statistics. To determine factors associated with attendance of research follow-up visits, generalized linear mixed effects models using a logistics link were fitted using repeated measures of exposure and primary outcome, including random intercepts for children and their families, since this study included some siblings from the same family. To analyze factors associated with time to withdrawal, Cox proportional hazard models with frailty terms for children and their families were fitted. For both analyses, two sets of models were fitted: separate models for each factor (Model 1) to examine the raw associations between each factor and the outcomes and an adjusted model combining all factors together in one model controlling for confounding and inferring potential causations (Model 2). All p-values were two-tailed and statistical significance was set at alpha = 0.05. The Bonferroni-adjusted alpha was 0.0022 (0.05 divided by 23 as we examined 23 factors) to adjust for multiple comparisons. R version 4.0.2 for Mac was used for all analyses [22].

## Results

**Fig 1:**
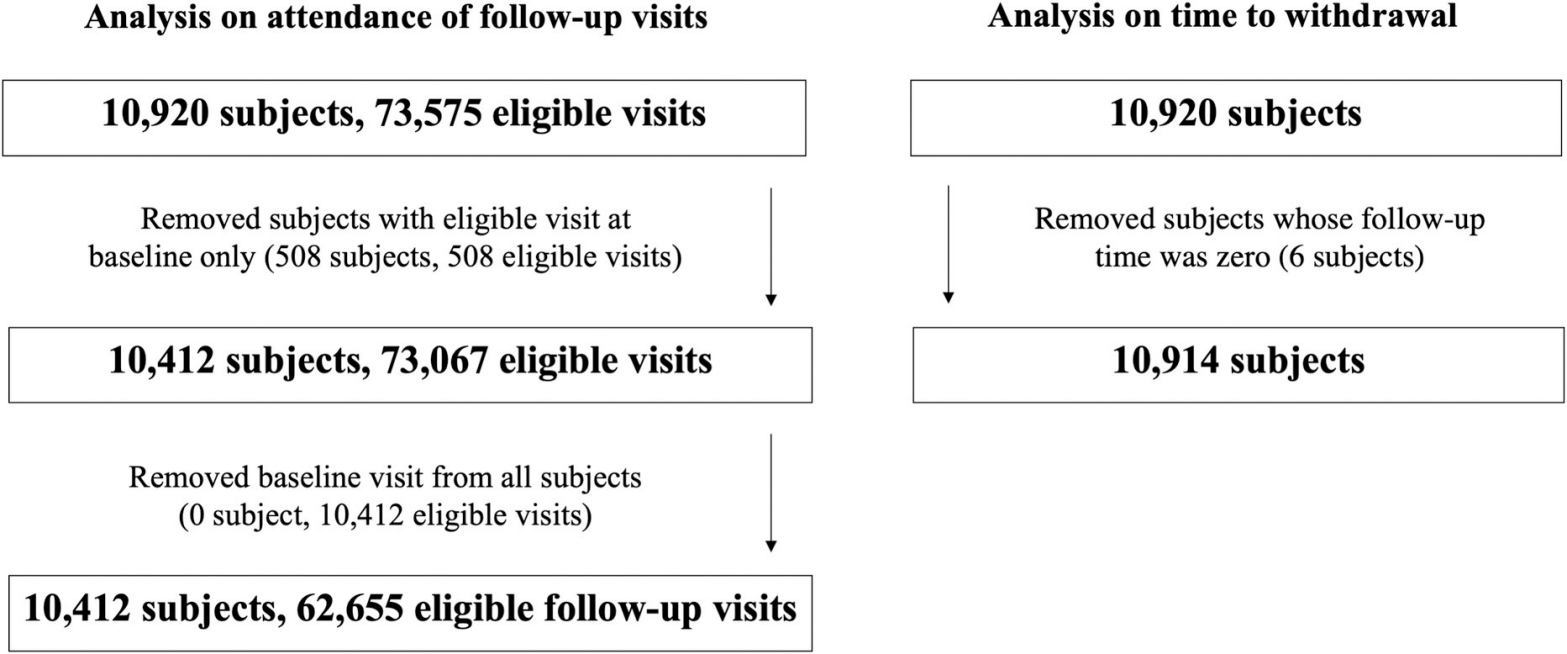
Sample size flow chart.

For the primary analysis, 10,412 children with 62,655 total eligible research follow-up visits were included between June 3, 2008 and March 4, 2020 (see **Fig 1** for sample size flowchart). Baseline, non-time-varying characteristics, collected at enrolment were presented in **Table 1**: the mean age at enrolment was 22.2 months, 52% were male, and 52% had mothers and fathers of European ethnicity. Time-varying characteristics were based on eligible research follow-up visits and are shown in **Table 2**: the majority of the families had $80,000 or more annual household income and 11.6% of the children had at least one chronic condition diagnosis. Study participation is described in **Table 3**. Out of the 62,655 eligible research follow-up visits, 31.2% were attended. Out of the 10,412 participants eligible for follow-up, 7,124 participants (68.4%) attended at least 1 research follow-up visit. Since 2008, 696 (6.4% out of 10,914) participants have submitted a withdrawal request and the mean time from enrolment to withdrawal was 3.5 years. Time-consuming was the main reason according to the research assistants’ documentation.

**Table 1:** Non-time-varying characteristics at enrolment.

N = 10,412	Level	Mean (SD) or N (%)
Family		8523
Child age at enrolment (months)		22.2 (19.2)
Child sex	Female	5001 (48.0)
	Male	5411 (52.0)
Maternal ethnicity	European	5423 (52.1)
	East Asian	609 (5.8)
	South Asian	858 (8.2)
	Southeast Asian	330 (3.2)
	Arab	201 (1.9)
	African	576 (5.5)
	Latin American	300 (2.9)
	Mixed ethnicity	540 (5.2)
	Other	31 (0.3)
	Missing	1544 (14.8)
Paternal ethnicity	European	5404 (51.9)
	East Asian	410 (3.9)
	South Asian	918 (8.8)
	Southeast Asian	256 (2.5)
	Arab	230 (2.2)
	African	731 (7.0)
	Latin American	237 (2.3)
	Mixed ethnicity	450 (4.3)
	Other	33 (0.3)
	Missing	1743 (16.7)
Maternal education level	College/University	8507 (81.7)
	Public/High school	917 (8.8)
	Missing	988 (9.5)
Maternal age at enrolment (years)^a^	<32	1841 (17.7)
	≥ 32 and < 35	2143 (20.6)
	≥ 35 and < 38	2345 (22.5)
	≥38	2759 (26.5)
	Missing	1324 (12.7)
Biological mother country of birth	Canada	5722 (55.0)
	Other	3594 (34.5)
	Missing	1096 (10.5)
Biological father country of birth	Canada	5459 (52.4)
	Other	3633 (34.9)
	Missing	1320 (12.7)
Child immigration status	Canadian citizen	8237 (79.1)
	International Adoptee/Landed immigrant/Refugee	106 (1.0)
	Missing	2069 (19.9)
Language spoken most often at home	English or French	4736 (45.5)
	Other	1034 (9.9)
	Missing	4642 (44.6)
Maternal employment	Yes (Full time/ part time/ on leave)	7351 (70.6)
	No	1946 (18.7)
	Missing	1116 (10.7)
Paternal employment	Yes (Full time/ part time/ on leave)	8624 (82.8)
	No	411 (3.9)
	Missing	1377 (13.2)
Child birthweight (kg)	< 2.5 kg	1007 (9.7)
	≥ 2.5 and < 4 kg	7615 (73.1)
	≥ 4 kg	927 (8.9)
	Missing	863 (8.3)
TARGet Kids! site at enrolment	Site 1	1502 (14.4)
	Site 2	1533 (14.7)
	Sites 3–7	42 (0.4)
	Site 8	198 (1.9)
	Site 9	1785 (17.1)
	Site 10	1061 (10.2)
	Site 11	26 (0.2)
	Sites 12–15	791 (7.6)
	Site 16	3 (0.03)
	Site 17	634 (6.1)
	Site 18	346 (3.3)
	Site 19	1910 (18.3)
	Site 20	581 (5.6)

^a^ Categorized based on quartiles (25^th^/50^th^/75^th^ percentile)

**Table 2:** Time-varying characteristics.

62,655 observations	Level	Mean (SD) or observation (%)
Age window of eligible research follow-up visit	0-6months	0 (0.0)
	9-15months	2842 (4.5)
	18months	5201 (8.3)
	2years	5721 (9.1)
	3years	5949 (9.5)
	4years	5989 (9.6)
	5years	6260 (10.0)
	6years	6220 (9.9)
	7years	5460 (8.7)
	8years	4822 (7.7)
	9years	4120 (6.6)
	10years	3469 (5.5)
	11years	2698 (4.3)
	12years	1895 (3.0)
	13years	1200 (1.9)
	14years	590 (0.9)
	15years+	219 (0.3)
Self-reported annual family income	less than $39,999	4971 (7.9)
	$40, 000 to $79, 999	6720 (10.7)
	$80, 000 to $149, 999	14094 (22.5)
	$150, 000 or more	22579 (36.0)
	Missing	14252 (22.7)
	Question not asked	39 (0.1)
Number of siblings	0	17828 (28.5)
	1	29505 (47.1)
	≥2	11133 (17.8)
	Missing	4189 (6.7)
Child living arrangement	Lives with 2 parents in the same household	54812 (87.5)
	Other	5180 (8.3)
	Missing	2663 (4.3)
Food insecurity ^a^	No	30813 (49.2)
	Yes	1621 (2.6)
	Question not asked	11430 (18.2)
	Missing	18791 (30.0)
Dwelling type	Apartment	6299 (10.1)
	House	30837 (49.2)
	Missing	17304 (27.6)
	Question not asked	8215 (13.1)
Child weight status ^b^	Underweight (zBMI < -2)	1543 (2.5)
	Normal weight (-2 ≤ zBMI ≤ 1)	48223 (77.0)
	Overweight (1< zBMI ≤ 2)	8947 (14.3)
	Obese/ severe obese (zBMI > 2)	2986 (4.8)
	Missing	956 (1.5)
Parent weight status ^c^	Underweight (BMI < 18.5)	1075 (1.7)
	Normal weight (18.5 ≤ BMI < 25)	31422 (50.1)
	Overweight (25 ≤ BMI < 30)	17898 (28.6)
	Obese (BMI ≥ 30)	9336 (14.9)
	Missing	2924 (4.7)
Parental diagnosis of chronic health conditions ^d^	Any	18130 (28.9)
	None	22960 (36.6)
	Missing	21565 (34.4)
Child diagnosis of chronic health conditions ^e^	Any	7282 (11.6)
	None	17673 (28.2)
	Missing	37700 (60.2)
Month of eligible research follow-up visit	January—April	19894 (31.8)
	May—August	21594 (34.5)
	September—December	21167 (33.8)

^a^ Food insecurity is classified as No if they answered “Never True” for the following 2 questions, otherwise food insecurity is classified as “Yes”:

• Within the past 12 months we worried whether our food would run out before we got money to buy more (Never true/ sometimes true/ often true)

• Within the past 12 months the food we bought just didn’t last and we didn’t have money to get more (Never true/ sometimes true/ often true)

^b^ zBMI: age- and sex- standardized body mass index (BMI) based on the World Health Organization (WHO) Child Growth Standards. BMI was calculated using height/length and weight, measured by a trained RA. According to the WHO weight status cut-offs for children, zBMI < -2 is classified as underweight, -2 ≤ zBMI ≤ 1 is classified as normal weight, 1 < zBMI ≤ 2 is classified as at risk for overweight for children < 5 year, overweight for children ≥5 year, zBMI > 2 is classified as overweight or obese for children < 5 year, obese or severely obese for children ≥5 year

^c^ BMI of the parent who accompanied their child at the visit. BMI was calculated using height/length and weight, measured by a trained RA. In adults, BMI < 18.5 is classified as underweight, 18.5 ≤ zBMI < 25 is classified as normal weight, 25 ≤ zBMI < 30 is classified as overweight, BMI ≥ 30 is classified as obese.

^d^ Maternal or paternal diagnosis of multiple sclerosis, diabetes, osteoporosis, heart disease, hypertension, high cholesterol, cancer, asthma, depression or anxiety, stroke, alcohol or drug problems, attention deficit hyperactivity disorder (ADHD), autism spectrum disorder (ASD), learning disability, overweight or obesity.

^e^ Child diagnosis of ADHD, allergies, asthma, ASD, developmental delay, diabetes, eczema, learning problem, overweight, cancer, or inflammatory bowel disease

**Table 3:** Attendance of research follow-up visits and withdrawal from TARGet Kids!.

	N (%) or Mean (SD)
**Total missed research follow-up visits**	43094 (68.8%)
**Total attended research follow-up visits**	19561 (31.2%)
**Number of attended research follow-up visits per subject**	
0	3288 (31.6%)
1 or 1+	7124 (68.4%)
**Participants who have submitted a withdrawal request**	696/10914 = 6.4%
**Time to withdrawal (years)**	3.5 (2.4)
**Research assistant-reported reasons for withdrawal**	
Inclusion/Exclusion criteria not fulfilled	8 (1.1%)
Lost to follow-up	30 (4.3%)
Patient withdrawal—time consuming	336 (48.3%)
Patient withdrawal—left the practice	150 (21.6%)
Death	2 (0.3%)
Other (Free text)	170 (24.4%)
Theme 1: No reason given	83 (11.9%)
Theme 2: Not interested	30 (4.3%)
Theme 3: Issues with bloodwork	27 (3.9%)
Other	30 (4.3%)

Results on factors associated with attendance of research follow-up visits are shown in **Table 4**. In the adjusted model (Model 2), older children, certain TARGet Kids! sites, families with lower income (less than $39,999 and $40,000 to $79,999 relative to $150,000 or more), and children living in an apartment had higher odds of missing a research follow-up visit compared to the reference groups; older mothers at enrolment (≥38 years relative to <32 years), children with siblings, and children with chronic health conditions had lower odds of having a missed research follow-up visit compared to the reference groups (see odds ratios, confidence intervals and p-values in **Table 4**). Child immigration status was not included in the model due to its low variability. Missingness was consistently associated with higher odds of having a missed research follow-up visit.

**Table 4:** Factors associated with missed research follow-up visits (10,412 subjects, 62,655 eligible follow-up visits).

Characteristics		Model 1 ^a^		Model 2 ^b^	
		OR (95%CI)	P-value	OR (95%CI)	P-value
Age window of eligible research follow-up visit	0–18 months	Ref			
	2–3 years	1.33 (1.24; 1.43)	**< .001**	1.78 (1.65; 1.93)	**< .001**
	4–5 years	1.90 (1.76; 2.04)	**< .001**	3.24 (2.97; 3.54)	**< .001**
	6–7 years	3.19 (2.94; 3.45)	**< .001**	6.27 (5.65; 6.97)	**< .001**
	8–9 years	4.83 (4.43; 5.28)	**< .001**	12.04 (10.58; 13.7)	**< .001**
	10 years and up	15.42 (13.93; 17.08)	**< .001**	49.17 (41.85; 57.76)	**< .001**
Child sex	Male	Ref			
	Female	1.02 (0.96; 1.08)	0.56	1.01 (0.95; 1.06)	0.83
Maternal ethnicity	European	Ref			
	East Asian	0.83 (0.73; 0.96)	0.01	0.82 (0.71; 0.95)	0.01
	South/Southeast Asian	1.87 (1.67; 2.10)	**< .001**	1.16 (0.99; 1.35)	0.06
	African	2.74 (2.33; 3.22)	**< .001**	1.07 (0.88; 1.30)	0.50
	Mixed ethnicity	1.11 (0.96; 1.29)	0.15	1.04 (0.91; 1.20)	0.56
	Latin American, Arab, and Other	1.63 (1.40; 1.90)	**< .001**	1.06 (0.89; 1.25)	0.53
	Missing	2.35 (2.12; 2.60)	**< .001**	1.50 (1.27; 1.78)	**< .001**
Paternal ethnicity	European	Ref			
	East Asian	0.85 (0.72; 1.00)	0.05	0.97 (0.82; 1.16)	0.76
	South/Southeast Asian	1.84 (1.64; 2.07)	**< .001**	1.00 (0.86; 1.17)	0.97
	African	2.44 (2.12; 2.81)	**< .001**	1.28 (1.08; 1.52)	0.004
	Mixed ethnicity	1.03 (0.88; 1.20)	0.75	1.02 (0.88; 1.17)	0.81
	Latin American, Arab, and Other	1.76 (1.50; 2.07)	**< .001**	1.32 (1.10; 1.57)	0.002
	Missing	2.04 (1.87; 2.26)	**< .001**	0.94 (0.80; 1.09)	0.40
Maternal education level	College/University	Ref			
	Public/High school	2.24 (1.99; 2.52)	**< .001**	0.99 (0.88; 1.12)	0.89
	Missing	48.92 (35.53; 67.35)	**< .001**	2.26 (1.51; 3.37)	**< .001**
Maternal age at enrolment (years)	<32	Ref			
	≥ 32 and < 35	0.63 (0.57; 0.70)	**< .001**	0.91 (0.83; 1.01)	0.07
	≥ 35 and < 38	0.61 (0.55; 0.67)	**< .001**	0.86 (0.78; 0.95)	0.004
	≥38	0.67 (0.60; 0.74)	**< .001**	0.77 (0.70; 0.85)	**< .001**
	Missing	1.68 (1.48; 1.92)	**< .001**	0.79 (0.66; 0.95)	0.01
Biological mother country of birth	Born in Canada	Ref			
	Not born in Canada	1.66 (1.55; 1.79)	**< .001**	1.06 (0.98; 1.15)	0.16
	Missing	3.55 (3.12; 4.04)	**< .001**	0.92 (0.68; 1.24)	0.58
Biological father country of birth	Born in Canada	Ref			
	Not born in Canada	1.64 (1.52; 1.76)	**< .001**	0.99 (0.92; 1.08)	0.86
	Missing	3.10 (2.77; 3.48)	**< .001**	0.79 (0.63; 0.99)	0.04
Language spoken most often at home	English or French	Ref			
	Other	1.87 (1.65; 2.12)	**< .001**	0.94 (0.82; 1.07)	0.34
	Missing	1.73 (1.62; 1.85)	**< .001**	0.66 (0.60; 0.73)	**< .001**
Maternal employment	Yes	Ref			
	No	1.56 (1.43; 1.70)	**< .001**	0.88 (0.81; 0.96)	0.004
	Missing	3.24 (2.84; 3.68)	**< .001**	1.11 (0.85; 1.44)	0.44
Paternal employment	Yes	Ref			
	No	1.87 (1.59; 2.21)	**< .001**	1.05 (0.90; 1.23)	0.54
	Missing	2.66 (2.38; 2.98)	**< .001**	1.38 (1.12; 1.69)	0.002
Child birthweight	< 2.5 kg	1.04 (0.94; 1.15)	0.43	0.95 (0.86; 1.05)	0.35
	≥ 2.5 and < 4 kg	Ref			
	≥ 4 kg	1.09 (0.99; 1.21)	0.09	1.15 (1.04; 1.28)	0.005
	Missing	1.79 (1.59; 2.02)	**< .001**	0.93 (0.80; 1.07)	0.32
TARGet Kids! site at enrolment	Site 19	Ref			
	Site 1	1.54 (1.39; 1.72)	**< .001**	1.00 (0.90; 1.11)	0.98
	Site 2	1.02 (0.91; 1.14)	0.69	1.87 (1.68; 2.08)	**< .001**
	Sites 3–7, Sites 12–15, Site 11	5.20 (4.43; 6.10)	0.17	7.09 (5.98; 8.39)	**< .001**
	Site 8, Site 16, Site 18	1.13 (0.95; 1.34)	**< .001**	4.26 (3.58; 5.07)	**< .001**
	Site 9	0.73 (0.66; 0.82)	**< .001**	1.11 (1.00; 1.23)	0.05
	Site 10	9.71 (8.15; 11.56)	**< .001**	12.01 (9.84; 14.64)	**< .001**
	Site 17	1.25 (1.08; 1.46)	0.004	2.16 (1.86; 2.51)	**< .001**
	Site 20	0.64 (0.54; 0.76)	**< .001**	2.64 (2.20; 3.17)	**< .001**
Self-reported annual family income	less than $39,999	3.10 (2.79; 3.45)	**< .001**	1.66 (1.44; 1.92)	**< .001**
	$40, 000 to $79, 999	1.69 (1.55; 1.84)	**< .001**	1.19 (1.07; 1.32)	**< .001**
	$80, 000 to $149, 999	1.15 (1.08; 1.23)	**< .001**	1.10 (1.03; 1.18)	0.007
	$150, 000 or more	Ref			
	Missing/Question not asked	10.05 (9.22; 10.96)	**< .001**	4.92 (4.39; 5.52)	**< .001**
Number of Siblings	0	Ref			
	1	0.87 (0.82; 0.93)	**< .001**	0.75 (0.70; 0.8)	**< .001**
	≥2	0.97 (0.89; 1.06)	0.48	0.65 (0.59; 0.71)	**< .001**
	Missing	10.82 (9.10; 12.86)	**< .001**	1.50 (1.22; 1.84)	**< .001**
Child living arrangement	Lives with 2 parents in the same household	Ref			
	Missing/Other	3.41 (3.09; 3.76)	**< .001**	0.79 (0.70; 0.88)	**< .001**
Food insecurity	No	Ref			
	Yes	1.46 (1.26; 1.70)	**< .001**	0.79 (0.67; 0.93)	0.004
	Missing	5.89 (5.51; 6.30)	**< .001**	3.89 (3.53; 4.29)	**< .001**
	Question not asked	0.75 (0.71; 0.79)	**< .001**	0.56 (0.5; 0.63)	**< .001**
Dwelling type	House	Ref			
	Apartment	1.51 (1.38; 1.66)	**< .001**	1.34 (1.21; 1.48)	**< .001**
	Missing	5.45 (5.09; 5.84)	**< .001**	3.53 (3.24; 3.85)	**< .001**
	Question not asked	0.77 (0.72; 0.82)	**< .001**	0.80 (0.74; 0.88)	**< .001**
Child weight status	Underweight and normal weight	Ref			
	Overweight	0.96 (0.90; 1.03)	0.27	0.92 (0.86; 0.99)	0.03
	Obese/ severe obese	1.22 (1.08; 1.38)	**0.001**	0.88 (0.77; 1.00)	0.05
	Missing	109.44 (44.72; 267.83)	**< .001**	3.13 (2.60; 3.75)	**< .001**
Parent weight status	Underweight and normal weight	Ref			
	Overweight	1.19 (1.12; 1.27)	**< .001**	1.02 (0.96; 1.09)	0.47
	Obese	1.37 (1.26; 1.49)	**< .001**	1.02 (0.93; 1.11)	0.67
	Missing	5.70 (4.82; 6.73)	**< .001**	3.13 (2.60; 3.75)	**< .001**
Parent diagnosis of chronic health conditions	None	Ref			
	Any	0.92 (0.86; 0.97)	0.005	0.97 (0.91; 1.03)	0.28
	Missing	2.62 (2.46; 2.78)	**< .001**	2.89 (2.65; 3.16)	**< .001**
Child diagnosis of chronic health conditions	None	Ref			
	Any	0.92 (0.85; 0.99)	0.02	0.78 (0.72; 0.85)	**< .001**
	Missing	1.78 (1.69; 1.88)	**< .001**	1.49 (1.39; 1.60)	**< .001**
Month of research follow-up visit	September—December	Ref			
	January—April	1.08 (1.02; 1.14)	0.005	0.94 (0.88; 0.99)	0.03
	May—August	1.10 (1.04; 1.16)	**< .001**	0.95 (0.89; 1.00)	0.06

Abbreviations: OR, odds ratio. CI, confidence interval.

^a^ Separate model for each factor.

^b^ All factors in one model.

The Bonferroni-corrected alpha is 0.0022 after adjusting for multiple (23) comparisons

The time-to-withdrawal analysis included 10,914 children (see **Fig 1**). Results on factors associated with time to withdrawal are shown in **Table 5**. In Model 2, the estimated hazard of withdrawal was 0.95 times lower with each 1 month increase in child age (adjust HR = 0.95; 95%CI: 0.94, 0.95; p < .001) after adjusting for the other variables in the model.

**Table 5:** Factors associated with time to withdrawal (10,914 subjects).

Characteristics		Model 1 ^a^		Model 2 ^b^	
		HR (95%CI)	P-value	HR (95%CI)	P-value
Child age in months		0.96 (0.95; 0.97)	**< .001**	0.95 (0.94; 0.95)	**< .001**
Child sex	Male	Ref			
	Female	0.94 (0.81; 1.09)	0.40	0.96 (0.82; 1.13)	0.64
Maternal ethnicity	European	Ref			
	East Asian	0.74 (0.49; 1.11)	0.15	0.67 (0.41; 1.09)	0.11
	South/Southeast Asian	1.87 (0.87; 1.49)	0.34	0.99 (0.64; 1.53)	0.96
	African	1.14 (0.79; 1.67)	0.45	1.27 (0.83; 1.93)	0.52
	Mixed ethnicity	0.90 (0.61; 1.34)	0.62	0.90 (0.59; 1.38)	0.63
	Latin American, Arab, and Other	1.60 (1.14; 2.25)	0.007	1.47 (0.97; 2.25)	0.07
	Missing	1.36 (1.06; 1.75)	0.01	1.25 (0.80; 1.93)	0.32
Paternal ethnicity	European	Ref			
	East Asian	0.92 (0.60; 1.42)	0.71	1.15 (0.68; 1.96)	0.61
	South/Southeast Asian	1.18 (0.91; 1.55)	0.22	1.28 (0.83; 1.98)	0.27
	African	1.32 (0.96; 1.78)	0.08	1.27 (0.83; 1.93)	0.27
	Mixed ethnicity	0.63 (0.39; 1.01)	0.05	0.59 (0.36; 0.98)	0.04
	Latin American, Arab, and Other	1.52 (1.05; 2.21)	0.03	1.14 (0.74; 1.78)	0.55
	Missing	1.22 (0.97; 1.55)	0.09	0.92 (0.60; 1.40)	0.69
Maternal education level	College/University	Ref			
	Public/High school	1.29 (0.99; 1.68)	0.06	1.17 (0.87; 1.59)	0.30
	Missing	1.88 (1.39; 2.53)	**< .001**	1.83 (1.14; 2.96)	0.01
Maternal age at enrolment (years)	<32	Ref			
	≥ 32 and < 35	0.82 (0.64; 1.04)	0.11	0.91 (0.7; 1.17)	0.45
	≥ 35 and < 38	0.77 (0.60; 0.98)	0.04	1.01 (0.77; 1.31)	0.97
	≥38	0.63 (0.50; 0.81)	**< .001**	0.97 (0.74; 1.26)	0.81
	Missing	0.74 (0.54; 1.00)	0.05	0.70 (0.41; 1.19)	0.18
Biological mother country of birth	Born in Canada	Ref			
	Not born in Canada	1.21 (1.02; 1.45)	0.03	1.15 (0.92; 1.44)	0.23
	Missing	1.14 (0.84; 1.55)	0.57	0.78 (0.35; 1.72)	0.54
Biological father country of birth	Born in Canada	Ref			
	Not born in Canada	1.23 (1.03; 1.47)	0.02	1.04 (0.83; 1.31)	0.71
	Missing	1.12 (0.88; 1.51)	0.32	1.01 (0.58; 1.74)	0.98
Language spoken most often at home	English or French	Ref			
	Other	0.99 (0.65; 1.51)	0.96	0.88 (0.57; 1.36)	0.57
	Missing	1.58 (1.30; 1.92)	**< .001**	2.48 (1.94; 3.17)	**< .001**
Maternal employment	Yes (Full time/ part time/ on leave)	Ref			
	No	1.33 (1.09; 1.61)	0.004	1.19 (0.95; 1.49)	0.58
	Missing	1.12 (0.82; 1.53)	0.48	1.46 (0.87; 2.43)	0.15
Paternal employment	Yes (Full time/ part time/ on leave)	Ref			
	No	1.20 (0.84; 1.72)	0.32	1.11 (0.76; 1.62)	0.58
	Missing	1.17 (0.90; 1.51)	0.24	1.46 (0.87; 2.43)	0.15
Child birthweight	< 2.5 kg	1.07 (0.81; 1.41)	0.65	1.18 (0.89; 1.58)	0.25
	≥ 2.5 and < 4 kg	Ref			
	≥ 4 kg	0.92 (0.70; 1.20)	0.53	0.89 (0.67; 1.18)	0.41
	Missing	1.21 (0.91; 1.61)	0.20	1.24 (0.87; 1.75)	0.23
TARGet Kids! site at enrolment	Site 19	Ref			
	Site 1	0.26 (0.19; 0.36)	**< .001**	0.21 (0.15; 0.29)	**< .001**
	Site 2	0.75 (0.59; 0.96)	0.02	0.74 (0.57; 0.97)	0.03
	Sites 3–7, Sites 12–15, Site 11	0.55 (0.37; 0.81)	0.003	0.38 (0.24; 0.59)	**< .001**
	Site 8, Site 16, Site 18	0.08 (0.03; 0.25)	**< .001**	0.14 (0.04; 0.44)	**< .001**
	Site 9	0.75 (0.60; 0.94)	0.01	1.02 (0.80; 1.31)	0.86
	Site 10	0.28 (0.17; 0.46)	**< .001**	0.20 (0.10; 0.37)	**< .001**
	Site 17	0.17 (0.09; 0.31)	**< .001**	0.15 (0.08; 0.29)	**< .001**
	Site 20	1.69 (1.15; 2.49)	0.007	2.03 (1.30; 3.18)	**< .001**
Self-reported annual family income	less than $39,999	1.46 (1.03; 2.07)	0.04	1.14 (0.76; 1.72)	0.53
	$40, 000 to $79, 999	1.66 (1.23; 2.24)	**< .001**	1.59 (1.15; 2.19)	0.005
	$80, 000 to $149, 999	1.29 (1.00; 1.68)	0.05	1.24 (0.95; 1.61)	0.12
	$150, 000 or more	Ref			
	Missing/Question not asked	2.40 (1.93; 2.98)	**< .001**	3.24 (2.41; 4.36)	**< .001**
Number of Siblings	0	Ref			
	1	0.84 (0.70; 1.00)	0.06	1.04 (0.86; 1.26)	0.70
	≥2	0.90 (0.70; 1.16)	0.42	1.22 (0.93; 1.61)	0.15
	Missing	1.17 (0.86; 1.57)	0.31	0.73 (0.49; 1.09)	0.13
Child living arrangement	Lives with 2 parents in the same household	Ref			
	Missing/Other	1.17 (0.93; 1.48)	0.18	1.04 (0.76; 1.43)	0.81
Food insecurity	No	Ref			
	Yes	1.03 (0.59; 1.79)	0.91	0.93 (0.52; 1.66)	0.80
	Missing	1.28 (1.05; 1.56)	0.02	0.86 (0.63; 1.18)	0.36
	Question not asked	1.20 (0.96; 1.49)	0.11	0.80 (0.56; 1.13)	0.20
Dwelling type	House	Ref			
	Apartment	1.51 (1.16; 1.94)	0.002	1.25 (0.93; 1.68)	0.13
	Missing	1.40 (1.13; 1.67)	0.001	1.12 (0.88; 1.44)	0.35
	Question not asked	1.26 (0.99; 1.62)	0.06	1.05 (0.79; 1.40)	0.73
Child weight status	Underweight and normal weight	Ref			
	Overweight	1.16 (0.94; 1.42)	0.16	1.17 (0.95; 1.44)	0.15
	Obese/ severe obese	1.04 (0.73; 1.48)	0.82	1.05 (0.72; 1.53)	0.81
	Missing	0.83 (0.44; 1.56)	0.57	0.53 (0.27; 1.05)	0.07
Parent weight status	Underweight and normal weight	Ref			
	Overweight	1.00 (0.83; 1.22)	0.97	0.90 (0.73; 1.10)	0.29
	Obese	1.19 (0.94; 1.51)	0.15	1.05 (0.82; 1.36)	0.68
	Missing	1.30 (0.94; 1.80)	0.11	1.24 (0.86; 1.79)	0.26
Parent diagnosis of chronic health conditions	None	Ref			
	Any	0.99 (0.79; 1.24)	0.95	1.03 (0.82; 1.30)	0.80
	Missing	1.37 (1.13; 1.67)	**0.001**	0.99 (0.74; 1.31)	0.93
Child diagnosis of chronic health conditions	None	Ref			
	Any	0.77 (0.56; 1.05)	0.09	0.85 (0.62; 1.16)	0.30
	Missing	1.04 (0.86; 1.25)	0.66	1.02 (0.78; 1.35)	0.88
Month of research follow-up visit	September—December	Ref			
	January—April	1.01 (0.83; 1.23)	0.89	0.97 (0.79; 1.19)	0.76
	May—August	1.11 (0.92; 1.34)	0.28	0.99 (0.82; 1.19)	0.90

Abbreviations: HR, hazard ratio. CI, confidence interval.

^a^ Separate model for each factor.

^b^ All factors in one model.

The Bonferroni-corrected alpha is 0.0022 after adjusting for multiple (23) comparisons

## Discussion

In this primary care practice-based pediatric longitudinal cohort, research participation was described, and we investigated multiple sociodemographic, health, and study design factors and their association with research participation. To our knowledge, our study is one of the first primary care cohort studies of children to examine factors associated with follow-up participation and withdrawal using a sophisticated methodological approach beyond descriptive statistics. It is worth noting that, a missed research visit could be due to research assistants not being on site, or unable to approach multiple participants at the well-child visit or other administrative reasons that we were unable to measure in this study.

When we examined each factor’s association with follow up, children with mothers with lower education level, unemployed parents, and lower household income were more likely to have a missed research follow-up visit. Our findings align with other pediatric cohort studies [4, 5, 8, 10, 11] and intervention programs [3, 23–25] demonstrating low socioeconomic status as a key attrition determinant. Consistent with previous literature, our study demonstrated that older children, [3, 11] ethnic minority groups, [4] younger mothers, [5, 8] parents born in a foreign country, [5, 10, 11] and children not living with 2 parents in the same household [3, 10] were independently associated with lower level of research participation. Our study showed that children and parents diagnosed with chronic health conditions had lower odds of having a missed research follow-up visit, suggesting that families with chronic health conditions may be more interested in participating in health-related research as they may be more concerned about their families’ health. This is a novel finding of our study. In the multivariable analysis, family income remained as a key attrition determinant, with a clear dose-response relationship between income and odds of missing a research follow-up visit. Although the participation of TARGet Kids! had no direct financial burden to the household, parents from low-income families may work multiple part-time jobs, making them difficult to commit to questionnaire completion. In addition to time demands, literacy demands and health and life stresses may also be barriers hindering research participation from low-income families [26]. Missingness in questionnaire data was also a key factor in both adjusted and unadjusted models, suggesting that individuals who did not complete the questionnaires were less likely to participate in future research visits [10].

In line with a large multi-centre children’s cohort study in Europe, [10, 11] children and parents with a higher weight status were more likely to miss a follow up visit. Although obesity is considered by many a chronic disease, individuals with obesity may not feel comfortable participating in a cohort study which involves having their weight and height measured by a research assistant in practice. This may be related to ongoing weight bias in practice, as well as stigma and discrimination towards children and parents with obesity [27] in the healthcare system. Alternatively, weight status may be confounded by other social determinants of health; after adjusting for income, education, and ethnicity, the association with weight status was no longer significant in the multivariable model [28, 29].

Various engagement and retention strategies have been utilized by longitudinal cohort studies to improve study retention. A systematic review [30] identified 95 strategies and classified them into 4 themes: reducing barriers to participation, creating a project community, follow-up/reminder strategies, and tracing strategies. TARGet Kids! has recently implemented multiple strategies to improve retention. We have established a parent advisory group called the Parent and Clinician Team (PACT), [18] composed of multiple parents (both mothers and fathers) from different families with diverse sociodemographic backgrounds, to partner with us. Since the establishment of the PACT, we have engaged PACT members in all stages of our research [31, 32]. Ongoing collaboration with the PACT has ensured that our approach to families, measures, and follow-up procedures are sensitive to the needs of families facing socioeconomic barriers and children from racialized communities. We also have a Patient and Family Engagement Specialist working with TARGet Kids! as a core team member, providing ongoing communication and support for the team to address parent and clinician perspectives, and creating opportunities to engage underrepresented populations. The PACT participated in all stages of this study including setting priorities, determining research questions, and interpreting and communicating findings.

The use of social media as a tool to promote research participation is increasing and has shown effectiveness in a number of observational studies and clinical trials, including those with hard-to-reach population [33, 34]. Effective use of social media has also been a focus of our retention strategies. Informed by the PACT, we have recently enhanced the TARGet Kids! website (www.targetkids.ca), Instagram, and Twitter account, creating a brand identity (e.g., study logo, colour palettes, and typography) to engage study participants. Communication tools such as infographics, newsletters, and parent-friendly summaries of our publications are also among the strategies used to engage parents, families and retain participants.

Our findings and input from the PACT members suggested a need for more tailored support and targeted strategies for families. In this study, families speaking a language other than English or French at home were less likely to have ongoing research follow-up visits. To reduce barriers for these families, we propose to adjust our inclusion criteria and implement translation services to translate TARGet Kids! consent forms and questionnaires into more used languages other than English or French among TARGet Kids! participating families. Families who report ethnicity other than European were more likely to miss a visit or withdraw participation in TARGet Kids!. We plan to ensure our staff is representative of the study population and implement cultural sensitivity training program focusing on Equity, Diversity, and Inclusion (EDI). Since families who withdrew from TARGet Kids! found that participation in the study was time-consuming, we have asked PACT members for feedback on questionnaires. We have reviewed our tools, removed overlapping questions, and started to administer shorter tools in more infrequent time intervals, to reduce respondent burden on participating families. Since families leaving TARGet Kids! participating practices was another reason for withdrawal, measures could be developed to follow these participants through online questionnaires, health insurance numbers, and at-home parent measurement for height and weight using standardized techniques. Furthermore, a more consistent approach for reimbursement for time for participation (gift cards distribution) can be implemented to ensure participants are compensated punctually, and incentives may be offered to participants who complete all data collection points [35]. Lastly, a child-friendly reward program (e.g., stickers, colouring books, or puzzle pieces for bloodwork completion) could be implemented to motivate children and their families.

Strengths of this study include the longitudinal study design with 12 years of follow-up, a standardized data collection procedure, a large sample size of over 10,000 children allowing us to collect repeated measures on multiple participant characteristics, and a sophisticated methodological approach to study research participation. Moreover, ongoing collaboration with the PACT has ensured that our study was well-informed by participating parents. There are potential limitations to our study. First, most of our data were parent-reported, which may have introduced self-reporting bias and may be limited to parents’ perspectives; research assistants’ perspectives were not described in this study. Second, other factors may have contributed to research participation but were not included in the model (e.g., full-time vs. part-time research assistant at each site, workload of the research assistant, blood draw participation, family involvement in other research studies). Although we provided adjustment for multiple testing, type I error may have still occurred. Lastly, results of this study may only be applicable to healthcare-based studies since our study population was predominantly recruited from primary care settings in urban areas of Canada.

## Conclusions

In this large primary care practice-based cohort study of children, we identified multiple factors associated with research participation. Our study confirmed that low-income families were less likely to have ongoing participation in research. Understanding determinants of attrition is crucial to inform and develop effective strategies to support target populations and improve study participation and completeness of retention data. Qualitative evidence from parents, research staff and health care providers may be helpful to further understand factors influencing parents’ decision-making around research participation to aid in the development of targeted retention strategies.
